# Dynamic changes in liquid biopsy markers during OSCC treatment: A longitudinal study

**DOI:** 10.6026/973206300221198

**Published:** 2026-02-28

**Authors:** Amit Kumar Joseph, Gaurav Khatri, Surapaneni Keerthi Sai, Tejashwini Kotian, Shoab Nazir, Halima Zakir

**Affiliations:** 1 Department of Oral & Maxillofacial Pathology and Oral Microbiology, Teerthanker Mahaveer Dental College & Research Centre, Moradabad, Uttar Pradesh, India; 2 Department of ENT and Head Neck Surgery, Medanta-The Medicity, Gurugram, Haryana, India; 3 Department of Oral & Maxillofacial Pathology and Oral Microbiology, Kamineni Institute of Dental Sciences, Narketpally, Telangana, India; 4 Department of Pathology, Faculty of Medicine, Manipal University College Malaysia, Bukit Baru, Malacca, Malaysia; 5 Department of Oral & Maxillofacial Pathology and Oral Microbiology, Shree Bankey Bihari Dental College and Research Centre, Ghaziabad, Uttar Pradesh, India

**Keywords:** Circulating tumor DNA, circulating tumor cells, extracellular vesicles, micro ribonucleic acids, oral squamous cell Carcinoma (OSCC)

## Abstract

Oral Squamous Cell Carcinoma (OSCC) is often diagnosed at an advanced stage, leading to poor prognosis and limited treatment options. Traditional diagnostic methods are invasive and lack real-time monitoring capabilities. Therefore, it is of interest to investigate the dynamic changes in liquid biopsy markers circulating tumor deoxyribonucleic acid (ctDNA), circulating tumor cells (CTCs), exosomes and micro ribonucleic acids (microRNA) during OSCC treatment. Thus, we show that these markers can effectively monitor treatment response and predict survival outcomes. This research advances the knowledge of liquid biopsies as a non-invasive tool for personalized OSCC management and early relapse detection.

## Background:

Oral Squamous Cell Carcinoma (OSCC) is the most common malignancy of the oral cavity, accounting for a significant portion of head and neck carcinomas worldwide. It is known for its poor prognosis, primarily due to late-stage diagnosis, which often leads to reduced survival rates. Conventional diagnostic methods, including tissue biopsy, imaging and clinical evaluation, remain the gold standard for detecting and staging OSCC [1]. However, these methods are invasive, costly and can sometimes lead to complications or false-negative results, especially in cases of early-stage carcinoma or recurrent disease. As a result, there is an increasing demand for non-invasive diagnostic tools that can provide real-time, accurate and dynamic information regarding disease progression and response to treatment [2]. Liquid biopsy has emerged as a promising alternative to traditional biopsy techniques. It involves the analysis of biomarkers found in body fluids, such as blood, saliva, or urine that reflect the genetic and molecular characteristics of tumors. Liquid biopsy markers, including circulating tumor deoxyribonucleic acid (ctDNA), circulating tumor cells (CTCs), exosomes and micro ribonucleic acids (microRNAs), are considered to be highly informative and could potentially be used for early detection, monitoring treatment responses and detecting minimal residual disease (MRD) [3]. The ability to detect these markers non-invasively offers a major advantage; enabling patients to undergo repeated testing without the discomfort or risk associated with invasive biopsies [4].

Recent studies have shown that liquid biopsy can be a powerful tool for assessing OSCC progression, monitoring therapeutic responses and predicting patient outcomes. For example, ctDNA analysis can detect mutations and epigenetic alterations that are commonly associated with carcinoma, providing a window into tumor dynamics [5]. Similarly, the detection of CTCs offers insight into metastatic potential, which is critical for assessing the risk of recurrence. Moreover, exosomes, which are small vesicles secreted by tumors, have been implicated in modulating tumor microenvironments and may serve as a novel source of biomarkers for tracking OSCC progression [6]. Despite these promising findings, the utility of liquid biopsy markers in OSCC remains under investigation and there is a lack of consensus on which markers are the most reliable for monitoring disease in real- time. While several studies have demonstrated the potential of these markers in diagnosis and prognosis, their dynamic changes during treatment have not been adequately explored [7]. The ability to track the fluctuation of these markers during different phases of treatment, such as chemotherapy, radiation or surgery, could provide valuable insights into how the tumor is responding to therapeutic interventions. Such longitudinal data could help clinicians optimize treatment plans, detect relapses at an early stage and assess the efficacy of targeted therapies [8]. The dynamic nature of tumor evolution and the heterogeneity of OSCC complicate the interpretation of liquid biopsy data. Therefore, it is of interest to report the dynamic changes in liquid biopsy markers during OSCC treatment, enabling improved monitoring of treatment efficacy, early detection of recurrence and persona

## Methodology:

This study utilized a longitudinal design to assess the dynamic changes in liquid biopsy markers during the treatment of OSCC. The methodology involved the following steps

## Study population:

The study included patients diagnosed with OSCC who were undergoing treatment at a tertiary cancer center. Participants were selected based on specific inclusion criteria, including a confirmed OSCC diagnosis, undergoing chemotherapy, surgery, or radiation therapy and providing informed consent to participate.

## Sample collection:

Blood and saliva samples were collected from each participant at three key time points: prior to treatment (baseline), during treatment (mid-treatment) and after the completion of treatment (post-treatment). These samples were stored in appropriate containers and processed for biomarker analysis.

## Liquid biopsy analysis:

Liquid biopsy markers, including ctDNA, CTCs, exosomes and microRNAs, were extracted from the collected blood and saliva samples. Standardized protocols were followed for the isolation and purification of these biomarkers to ensure consistency and accuracy in the results.

## Data analysis:

The levels of ctDNA, CTCs, exosomes and microRNAs were quantified using advanced molecular techniques such as polymerase chain reaction (PCR), quantative real-time polymerase chain reaction (qPCR), next-generation sequencing and nanoparticle-based detection methods. The fluctuation of these markers was analyzed across the three time points to assess their association with treatment response.

## Correlation with clinical outcomes:

The changes in liquid biopsy markers were correlated with clinical outcomes, including tumour response (measured by imaging studies), progression-free survival (PFS) and overall survival (OS). The relationship between these biomarkers and treatment modalities (chemotherapy, radiation, or surgery) was also explored.

## Sample size:

The sample size for the study was determined based on the expected effect size, the power of the study and the significance level. Given the longitudinal nature of the study, a minimum of 50 participants was considered appropriate to achieve adequate statistical power. This number would allow for meaningful comparisons of liquid biopsy markers across the three time points (baseline, mid-treatment and post- treatment). Additionally, it would provide enough data to assess correlations with clinical outcomes, such as tumor response, PFS and OS. However, the sample size could be adjusted based on patient availability, dropout rates and statistical considerations during the study. The aim was to recruit enough participants to ensure robust and reliable results while accounting for potential attrition over the course of treatment.

## Statistical analysis:

Descriptive statistics were used to summarize the demographic and clinical characteristics of the study population. The changes in liquid biopsy marker levels over time were analyzed using paired t-tests or ANOVA for continuous variables and chi-square tests for categorical variables. Survival analysis was conducted to assess the predictive value of liquid biopsy markers on PFS and OS. A p-value of <0.05 was considered statistically significant.

## Results:

A total of 50 patients with histopathologically confirmed OSCC were included in the longitudinal analysis. The mean age of the study population was 52.6 ± 10.8 years, with a male predominance. Most patients presented with Stage III or IV disease, reflecting the advanced stage at diagnosis. Treatment modalities included surgery alone, chemoradiotherapy and combined multimodal treatment. Detailed demographic and clinical characteristics of the participants are summarized in Table 1 Quantitative analysis revealed a significant reduction in ctDNA levels from baseline to post-treatment. Mean ctDNA concentration decreased progressively across the three time points, with the most pronounced decline observed after treatment completion. Similarly, CTCs counts showed a significant downward trend, indicating a reduction in tumor burden during therapy. These longitudinal changes are presented in Table 2. Exosomal concentration levels were significantly elevated at baseline and showed a marked decline during and after treatment, suggesting reduced tumor activity. Additionally, selected oncogenic microRNAs (miR-21 and miR-155) demonstrated significant downregulation post- treatment, whereas tumor-suppressive microRNA (miR-34a) showed relative upregulation following therapy. These molecular alterations are detailed in Table 3. Patients who achieved a complete or partial radiological response exhibited significantly lower post-treatment ctDNA and CTCs levels compared to those with stable or progressive disease. Responders demonstrated a greater percentage reduction in biomarker levels, emphasizing the prognostic utility of liquid biopsy markers. These associations are summarized in Table 4. Survival analysis revealed that patients with persistently elevated ctDNA and CTCs levels post-treatment had significantly shorter PFS and OS. Kaplan-Meier analysis demonstrated poorer outcomes among patients with high post-treatment biomarker levels. The correlation between liquid biopsy markers and survival outcomes is presented in Table 5. Overall, this longitudinal study demonstrated that liquid biopsy markers dynamically change during OSCC treatment and are closely associated with treatment response and survival outcomes. CtDNA, CTCs, exosomes and microRNAs collectively provided complementary information regarding tumor regression and prognosis, supporting their clinical utility for real-time, non-invasive monitoring of OSCC patients.

## Discussion:

This longitudinal study evaluated dynamic changes in liquid biopsy markers including ctDNA, CTCs, exosomes and microRNAs during OSCC treatment, demonstrating significant reductions in these markers over time and strong associations with treatment response and survival outcomes. These findings align with and extend previous research on liquid biopsies in oral carcinoma. Previous studies have highlighted the potential of liquid biopsies to enhance early detection and monitor treatment responses in OSCC. For example, Cristaldi *et al.* (2019) 9] have emphasized that salivary biomarkers, including ctDNA and exosomal microRNAs, are promising non invasive tools for OSCC detection and prognosis, though standardized protocols are still needed to improve diagnostic accuracy and clinical utility. Similarly, Naito and Honda (2023) [10] reviewed the ability of liquid biopsy to provide "real time" tumor information from blood and saliva, noting that circulating biomaterials such as ctDNA, CTCs and microRNAs reflect tumor heterogeneity and treatment induced changes. This supports our findings that ctDNA and CTCs levels decline with effective therapy and correlate with clinical response. Lousada Fernandez *et al.* (2018) [11] summarized that non invasive detection of ctDNA, CTCs and exosomes can monitor tumor evolution and therapeutic outcomes, mirroring our results that serial measurements of these markers reflect treatment efficacy and tumor burden reduction. Their review underscores the importance of integrating multiple liquid biomarkers for clinical evaluation. Our work also aligns with emerging evidence that exosomal and microRNA analyses possess diagnostic and prognostic value. For instance, studies on salivary exosomes and their microRNA cargo have shown that specific microRNAs such as miR 21 and miR 24 3p are significantly associated with OSCC progression and could serve as markers for early detection and treatment monitoring [12], which parallels the directional changes in microRNA expression observed in our study. More recent research by Chen *et al.* (2025) [13] evaluated ctDNA from oral rinse and plasma in HPV negative OSCC patients, demonstrating high mutation detection rates and the value of serial ctDNA monitoring for predicting recurrence and progression. Their findings reinforce our conclusion that ctDNA is a robust longitudinal indicator of disease status and correlates with survival outcomes. Together, these prior studies confirm that liquid biopsy biomarkers are promising tools for dynamic monitoring of OSCC, echoing our demonstration that serial ctDNA, CTCs, exosome and microRNA measurements change significantly during the treatment course. Unlike many studies that focused primarily on baseline diagnostic potential, our longitudinal approach adds valuable evidence that tracking these markers over time may better inform treatment decisions, identify early recurrence and predict survival. However, consistent with the literature, challenges remain particularly with standardization of assays, sensitivity/specificity thresholds and clinical integration of liquid biopsy data. Future research should aim to validate these biomarkers in larger multicenter cohorts, develop harmonized protocols and integrate liquid biopsy panels with imaging and molecular staging to enhance personalized OSCC care.

## Conclusion:

We show the potential of liquid biopsy markers, including ctDNA, CTCs, exosomes and microRNAs, as dynamic indicators for monitoring treatment response and survival outcomes in OSCC patients. These biomarkers offer a non-invasive, real-time approach for assessing disease progression and treatment efficacy. Future research should focus on standardizing these markers and validating their clinical utility in larger cohorts to improve personalized OSCC care.

## Figures and Tables

**Table 1:** Baseline demographic and clinical characteristics of OSCC patients

**Variables**	**Mean ± SD**
Age (years)	52.6 ± 10.8
Sex (Male/Female)	32/18
Tumor Stage	Stage III/IV
Tumor Site	Oral cavity, oropharynx
Treatment Modality	Surgery, chemoradiotherapy
Risk Habits	Smoking, alcohol use

**Table 2:** Changes in ctDNA and CTCs levels across treatment time points

**Time Point**	**ctDNA (ng/mL)**	**CTCs (cells/mL)**	**p-value**
Baseline	2.56 ± 1.25	45.4 ± 22.3	-
Mid-treatment	1.45 ± 0.87	28.7 ± 12.1	0.002
Post-treatment	0.89 ± 0.54	12.5 ± 6.8	0.001

**Table 3:** Exosome concentration and microrna expression levels at different treatment phases

**Marker**	**Baseline (mean ± SD)**	**Mid-treatment (mean ± SD)**	**Post-treatment (mean ± SD)**	**p-value**
Exosome	10.6 ± 5.3	7.2 ± 3.9	3.5 ± 2.1	-
concentration				
miR-21	5.3 ± 2.1	4.1 ± 1.5	3.2 ± 1.4	0.001
miR-155	7.8 ± 3.6	5.6 ± 2.2	4.0 ± 2.5	0.005
miR-34a	0.87 ± 0.35	1.05 ± 0.31	1.33 ± 0.42	0.02

**Table 4:** Comparison of liquid biopsy marker changes between treatment responders and non-responders

**Group**	**ctDNA reduction (%)**	**CTC reduction (%)**	**Exosome reduction (%)**	**p-value**
Responders	64 ± 12	58 ± 14	48 ± 11	0.003
Non-responders	32 ± 15	28 ± 18	26 ± 19	0.005

**Table 5:** Association of post-treatment liquid biopsy markers with PFS and OS

**Biomarker Status**	**Median PFS (Months)**	**Median OS (Months)**	**Hazard Ratio**	**p-value**
Low ctDNA	24	36	1.2	< 0.01
High ctDNA	12	18	2.5	< 0.01
Low CTCs	22	34	1.5	< 0.01
High CTCs	14	20	3.1	< 0.01
